# Lung Microbiota and Ventilator-Associated Pneumonia in the Neonatal Period

**DOI:** 10.3390/pathogens13030220

**Published:** 2024-03-01

**Authors:** Fermín García-Muñoz Rodrigo, Lourdes Urquía Martí, Marta Siguero Onrubia, Moreyba Borges Luján, Gloria Galán Henríquez, Desiderio Reyes Suárez

**Affiliations:** Neonatal Division, Complejo Hospitalario Universitario Insular Materno-Infantil, 35016 Las Palmas de Gran Canaria, Spain; murqmar@gobiernodecanaras.org (L.U.M.); msigonr@gobiernodecanaras.org (M.S.O.); mborluj@gobiernodecanaras.org (M.B.L.); ggalhen@gobiernodecanaras.org (G.G.H.); dreysua@gobiernodecanaras.org (D.R.S.)

**Keywords:** broad-spectrum antibiotics, endotracheal tube, microbioma, microbiota, multidrug resistant pathogens, neonate, prebiotics, probiotics, ventilator-associated pneumonia, very-low-birth-weight infant

## Abstract

The lung microbiota is a complex community of microorganisms that colonize the respiratory tract of individuals from, or even before, birth. Although the lungs were traditionally believed to be sterile, recent research has shown that there is a diversity of bacterial species in the respiratory system. Knowledge about the lung microbiota in newborns and its relationship with bacterial infections is of vital importance to understand the pathogenesis of respiratory diseases in neonatal patients undergoing mechanical ventilation. In this article, the current evidence on the composition of the lung microbiota in newborns will be reviewed, as well as the risks that an altered microbiota can impose on premature newborns. Although advances in neonatal intensive care units have significantly improved the survival rate of preterm infants, the diagnosis and treatment of ventilator-associated pneumonia has not progressed in recent decades. Avoiding dysbiosis caused by inappropriate use of antibiotics around birth, as well as avoiding intubation of patients or promoting early removal of endotracheal tubes, are among the most important preventive measures for ventilator-associated pneumonia. The potential benefit of probiotics and prebiotics in preventing infectious, allergic or metabolic complications in the short or long term is not clearly established and constitutes a very important field of research in perinatal medicine.

## 1. Introduction

In adults, it is estimated that the most common healthcare-related infection is pneumonia, with approximately one-third of cases being related to mechanical ventilation [1]. Although mortality attributable to ventilator-associated pneumonia (VAP) is difficult to establish, it seems clear that this complication is accompanied by an increase in the duration of mechanical ventilation and the length of stay of patients in Intensive Care Units (ICU) [2]. However, the concept of VAP is complex and difficult to establish in clinical practice, which makes it difficult to standardize criteria and generalize them so that they are useful in clinical decision making, as well as for benchmarking purposes and in research (especially epidemiological research). Traditionally, clinical, radiological and microbiological criteria have been used in adults, such as those of the North American Centers for Disease Control and Prevention (CDC) [3]. However, its application in the neonatal period is not simple, and there are not many quality studies that have validated its usefulness [4].

In recent decades, advances in laboratory technology have allowed us to witness a shift of paradigm. It has been discovered that the lung, long considered a sterile organ, has its own microbiota that is established from the moment of birth, or even before. Under normal and balanced conditions, it seems to protect the health of the individual; in contrast, alterations in the normal colonization process could be the origin of different pathologies, both in the short and long term, at later stages of life. The development of metagenomics, which is the study of the structure and function of entire nucleotide sequences isolated and analyzed from all the organisms (typically microbes, such as those residing on the human skin, gut, lungs, etc.), has provided us with information about the functional characteristics of a given microbial community. It allows for obtaining genomic sequences of microorganisms without the need to cultivate them, extracting and analyzing their DNA without previous amplification. Techniques have been developed to exclude dead bacteria and avoid biased results, amplifying the DNA of the viable ones. However, it should be noted that bacterial viability is not required to induce or propagate inflammatory responses, so the identification of non-viable bacteria could also be clinically relevant [5]. All this has led to a change in direction in microbiology, due to its high performance and relatively low cost. Knowledge of the bacterial influence on cellular functioning in their corresponding niches may allow for the development of new and promising therapies. A comprehensive overview of the evolution of culture-based microbiological techniques toward culture-independent DNA sequencing techniques is beyond the scope of this review. For a detailed study of it, as well as to become familiar with all the related terminology (transcriptome, proteome, metabolome, etc.), see Sherman MP et al. [6].

## 2. The Lung Microbiota and Microbiome

The microbiota is the set of live microorganisms that develop in a specific environment, and they can be commensal, symbiotic, or pathogenic. It is estimated that the human body has a similar number of eukaryotic cells and microorganisms, most of which are found in the intestine, skin and oral cavity [7]. In turn, we use the term microbiome to refer to the complete set of microorganisms, their genes, and their biomolecules in a certain environment [8]. This could be highly variable depending on the geographical location, age, health status, etc. [9]. Alterations in the microbiome are called “dysbiosis”, and this situation can be associated with significant alterations in the individual’s health status [10]. As a general rule, it is accepted that greater diversity in the microbiota is related to greater chances of health, while its reduction could favor the development of diseases through various mechanisms. Diversity describes the number of different taxa within a community. In turn, the microbial variation in a sample is called “α diversity”, while the variation in microbial communities between samples is called “β diversity” [11,12].

The process of lung colonization in newborns is not fully understood. Technical and ethical difficulties have prevented its study in detail [4]. Saprophytic or commensal bacteria that usually colonize different human organs are not usually isolated in conventional cultures, and therefore their study proved elusive until the arrival of new DNA sequencing and transcriptomics methods, such as the sequencing of the variable regions of the 16S ribosomal RNA gene (16S-rRNA), the gene most frequently used for bacterial identification. On the other hand, access to proximal anatomical areas, such as the nasopharyngeal or oropharyngeal cavity, the anorectal tract or the vagina, is relatively simple. However, access to the distal airway without contamination by agents located in more proximal locations has always been a great challenge, mostly in smaller patients, such as the neonate. For this reason, the lungs were not initially included in the human microbiome project [13].

It is generally accepted that colonization patterns in newborns differ depending on the route of delivery [14], with the lung microbiota being more diverse in those born vaginally than in those born by Cesarean section. Furthermore, the predominant microbiota in babies born vaginally, both in the intestine and in the lungs, is usually related to the mother’s vaginal microbiota, while in babies born by Cesarean section, it follows the mother’s skin microbiota pattern. Other studies show, however, that pulmonary colonization of the newborn could begin in the womb [15,16,17], although not all authors agree that the placenta has its own microbiota [18]. Of note, Lal et al. showed similar microbiomes in the respiratory tract of newborns delivered vaginally or by Cesarean section, suggesting an acquisition of microbial DNA that likely occurred through the placenta [11]. Interestingly, this study also showed an established and similar respiratory microbiome at birth in preterm and full-term infants. Differences in methodology or target populations are probably the basis for these discrepancies. In any case, the debate continues. There is now a growing belief that there is an intrauterine microbiota that influences fetal programming and development. Prenatal stress modifies the maternal microbiome, which can lead to the translocation of bacteria to the intrauterine environment, provoking an inflammatory response and triggering premature birth [19]. Pammi et al., in their systematic review, conclude that microbial dysbiosis may be associated with the progression and severity of BPD in at-risk infants [20]. Furthermore, it is widely accepted that the composition of a newborn’s lung microbiota is dynamic and changes under local selective pressures, but also with age, the environment, the type of diet, presence of stress, etc. [9,19,20,21]. A large proportion of neonates are exposed to antibiotics administered to their mothers as prophylaxis against group B streptococcal disease, chorioamnionitis, premature rupture of membranes, etc. [22]. Many of them also continue to receive empiric antibiotics, such as ampicillin and gentamicin [23], after birth. Although these interventions may be instrumental in reducing infant mortality, they may also have adverse effects on the gut microbiota, with important repercussions occurring in the lungs in the short and long term [24]. In their experimental model, Stevens J. et al. demonstrated that early exposure to antibiotics disrupted the maturation of intestinal commensal bacteria and altered the development of the lung immune system, making newborn macaques more susceptible to bacterial pneumonia. Furthermore, early antibiotic-induced intestinal dysbiosis has been associated with an increased long-term risk of asthma and allergies [21].

Currently, the concept of the intestinal–pulmonary axis is gaining more and more interest and relevance [25,26,27]. Some studies suggest initial oropharyngeal and digestive colonization, with the respiratory system secondarily colonized by descending route or microaspirations [28]. In addition to a direct role in terms of the characteristics and variety of the microbiota of both organs or systems, there is evidence of a remote immunomodulatory effect through certain micrometabolites produced by intestinal bacteria [29]. Bacterial fermentation of dietary fibers generates metabolic products that act as local and systemic signaling molecules helping to maintain immune and tissue homeostasis. Among the most studied, short chain fatty acids (SCFA) exert immunomodulatory functions in the bone marrow, influencing immune cell generation and development [30]. Furthermore, the gut microbiota possesses a metabolic capacity that the human gastrointestinal tract does not, providing the host with additional nutrients and energy [31]. Colonic anaerobiosis is essential for the growth of a balanced community of SCFA-producing microbiota, mainly from the phyla Firmicutes and Bacteroidetes, and prevents the growth of dysbiotic bacterial communities, such as Proteobacteria [32].

On the other hand, some studies have related the use of H_2_ blockers to changes in the microbiota, both intestinal and respiratory, and this was clinically associated with an increased risk of late onset sepsis, necrotizing enterocolitis and pneumonia [33]. In fact, the use of probiotics and prebiotics has been proposed to prevent these complications, but so far the low to moderate evidence of their effects, together with the potential associated morbidity and mortality in very or extremely preterm neonates, makes it necessary for more large, high-quality clinical trials to provide evidence of the validity and applicability of such interventions [34]. In addition to modifying the susceptibility to certain infections, it is likely that the airway microbiota also influences the structural development of the lung at critical moments [35]. Finally, the interaction between the intestine and the lungs could be in both directions. Respiratory viral infections, such as respiratory syncytial virus or influenza, could alter the intestinal microbiota [36], increasing the risk of subsequent enteric infection [37], while influenza-induced intestinal microbial changes increase the susceptibility to secondary pneumococcal infection [38].

Regarding the type of organisms colonizing the respiratory tract, once again, the studies are methodologically diverse and there is no absolute certainty regarding what the “normal” microbiota is in a healthy newborn. Nevertheless, it is generally accepted that the most favorable microbiome would be the one produced after a vaginal birth and where the baby is breastfed by their own mother [39]; however, it is also recognized that there may be great interindividual variability [40]. The bacterial phyla and genera most frequently present in the human microbiota, both in health and disease, are summarized in Table 1. In healthy adults, the most frequently found bacteria correspond to the phylum *Bacteroidetes*, especially the genus *Prevotella*, and the phylum *Firmicutes*, mainly the genera *Streptococcus* and *Veillonella* [41].

In the upper airways of healthy full-term newborns, large numbers of *Staphylococcus* spp., and later *Corynebacterium* spp., as well as *Dolosigranulum* spp. can be found during the first week of life, which is associated with greater stability of the bacterial community and good respiratory health. These saprophytic bacteria can inhibit the growth of *Staphylococcus aureus* and *Streptococcus pneumoniae*, probably through the production of certain antimicrobial peptides [42]. In contrast, in babies born by Cesarean section, *Staphylococcus aureus* persists, and anaerobes appear, including *Prevotella*, *Veillonella*, and *Porphyromonas* spp. [43]. On the other hand, as mentioned above, breastfeeding protects against infections, either through the transmission of antibodies [44] or protective microorganisms, such as *Bifidobacterium* spp. or *Lactobacillus* spp. [45], along with certain oligosaccharides that favor their development [46]. On the contrary, the perinatal use of antibiotics can lead to a decrease in beneficial bacteria, such as *Dolosigranulum* spp. and *Corynebacterium* spp., and a higher proportion of potential pathogens such as *Haemophilus*, *Streptococcus* and *Moraxella* [47], increasing the risk of respiratory tract infections [48], and even bacterial resistance in the long term [49]. For a more in-depth discussion of the mechanisms that contribute to the establishment of a healthy respiratory microbiota, and the specific host-microbiota interactions that support it, as well as the interrelationship between bacteria, or between bacteria and viruses (virome) or fungi (mycobiome) also present in the human body, see the interesting and comprehensive review by Man WH et al. [50].

**Table 1:** Main phyla and genera of bacteria in the human microbiome, most of them present in human milk (Adapted from Jeurink PV et al. [45]).

Phyla	Actinobacteria	Bacteroidetes	Firmicutes	Proteobacteria	Tenericutes	Verrucomicrobia
***Genus***	*Bifidobacterium* *Corynebacterium* *Dermatobacter* *Kocuria* *Mycobacterium* *Parascovia* *Propionibacterium* *Rothia*	*Bacteroides* *Prevotella* *Porphyromonas* *Rikenella*	*Bacillus* *Clostridium* *Dolosigranulum* *Enterococcus* *Lactobacillus* *Leuconostoc* *Pediococcus* *Staphylococcus* *Streptococcus* *Veillonella* *Weisella*	*Acinetobacter* *Bradyrhizobiaceae* *Burkholderia* *Escherichia* *Haemophilus* *Helicobacter* *Moraxella* *Neisseria* *Novosphingobium* *Pateurella* *Pseudomonas* *Ralstonia* *Serratia* *Sphingobium* *Sphingomonas* *Sphingopyxis*	*Mycoplasma* *Ureaplasma*	*Akkermansia*

In premature babies at risk of bronchopulmonary dysplasia, bronchial aspirates showed an increase in pathogens such as *Staphylococcus aureus*, *Pseudomonas aeruginosa* and *Streptococcus* spp. [51] or *Ureaplasma* spp. [52]. It has also been suggested that bacterial diversity in intubated patients decreases during antibiotic treatment [53]. This could increase the development of pneumonia due to resistant pathogens that may be present in biofilms that develop in endotracheal tubes [54]. On the other hand, the communication mechanisms between cells and tissues in an organism at various levels (hormones, neurotransmitters, citokines, etc.) can produce cross-talk with the world of microbes [55]. This knowledge has led to the development of the “microbial endocrinology” [56]. An example of these interactions could be the significant growth demonstrated in vitro of Pseudomonas aeruginosa in the presence of catecholamines (norepinephrine and dopamine) [57]. However, to date, no study has evaluated whether exogenous catecholamines independently influence the lung microbiome.

In any case, accurately specifying the source of the microbiota in the newborn, as well as its evolution as the individual develops and is exposed to other environmental factors, would require detailed sequencing of the genome of organisms from multiple locations, not only from the newborn itself, but also from the birth canal, the mother skin and breast milk, the skin of the health personnel who assist the birth, the ecology of the units where patients are admitted, etc. [21].

## 3. Ventilator-Associate Pneumonia (VAP)

### 3.1. Definition and Epidemiology

VAP is an inflammation of the lung produced by infectious agents that were not present or in incubation when the patient was started on mechanical ventilation (MV). Clinically, it is defined as a nosocomial lung infection diagnosed in patients undergoing MV for at least 48 h. It is considered the second most common cause of nosocomial infection in neonatal and pediatric intensive care patients, and it is estimated that its incidence ranges between 1 and 63 episodes per 1000 days of ventilation, depending on the degree of development of the countries [58]. Apart from differences in patients and unit characteristics, this variability in incidence of VAP could also be explained by the use of different diagnostic criteria to define VAP. Among the factors that favor abnormal bacterial colonization and lung infections in newborns are, apart from the immaturity of the patient’s immune system, pre- and postnatal malnutrition, naso- or orotracheal intubation and reintubations, and, above all, the duration of mechanical ventilation, the use of previous antibiotics, and the environmental ecology of the units [59,60]. Of all of them, prolonged intubation is the one that has been most independently associated with the appearance of VAP [61]. Intubation alters the natural lung defense mechanisms, such as ciliary movements to clear mucus or coughing. Furthermore, tubes surpass the glottis and the larynx, connecting the oropharynx with the pulmonary ecosystem, altering the abundance and composition of the microbiota [62]. Bacterial biofilms form on the walls of the tubes, protecting them from antibiotics and the immune system [63].

According to the CDC, VAP is identified through a combination of imaging, clinical, and laboratory criteria, and it is considered ventilator-associated when the patient is on mechanical ventilation for >2 consecutive days at the time of the event and the ventilator was in place on the day of the event or the day before [3]. Table 2 is an adaptation of the CDC clinical and radiological criteria for patients ≤ 12 months of age. These criteria allow for the diagnosis of “clinically defined pneumonia” even without pathogen isolation, given the difficulty in obtaining uncontaminated samples from the respiratory tract of infants. However, some authors have emphasized the importance of microbiological diagnosis to avoid overdiagnosis of VAP and excessive use of antibiotics [64]. On the contrary, the isolation of pathogens without clinical and radiological signs could simply represent a colonization of the respiratory tract. In short, the current CDC definitions are not specific for the neonatal population, and even less so for the group of very-low-birth-weight (VLBW) infants. Isolated positive tracheal culture alone does not distinguish between bacterial colonization and respiratory infection [60]. Clinical and laboratory signs are generally nonspecific and may correspond to other conditions, such as bronchopulmonary dysplasia and nosocomial sepsis. Radiological reports in infants with airway colonization without definitive clinical and laboratory evidence of infection could be misleading [65]. All of this reflects the difficulty in establishing homogeneous and universal diagnostic criteria for neonatal VAP. The inter-observer variability, as well as the absence of a “gold or reference standard”, make the precision of the diagnoses weak and studies difficult to compare. For certain clinical conditions, in addition to the clinical and radiological criteria, the CDC offers microbiological criteria for the diagnosis of VAP that are summarized in Table 3.

Given the lack of consensus regarding the definition of VAP in neonates and the low reliability of chest X-rays, a lung ultrasound is now considered a potential alternative diagnostic tool. A recent study by Tusor et al. [66] showed that a multiparameter VAP score combining clinical, microbiology, and a lung ultrasound improved the sensitivity, specificity, and the area under curve for VAP detection in preterm infants with chronic lung disease in comparison with clinical information alone or the combination of clinical information plus chest-X-ray. In 2013 for adults [67], and in 2019 for pediatric and neonatal patients [68], the National Healthcare Safety Network (NHSN) of the CDC replaced the criteria of VAP surveillance with surveillance of ventilator-associated events (VAEs), defined primarily as an increase in respiratory support (oxygen or pressure) in a previously stable or improving mechanically ventilated patient. The aim was to obtain a more general and objective criterion of complications associated with the ventilator to detect both infectious and non-infectious complications. These VAE definitions have the necessary validity and reliability to be used in external quality assessments and benchmarking [69]. However, VAP definitions, despite the difficulties described, have been shown to be useful as a measure of improving the internal quality of centers on an individual level [70].

### 3.2. Sample Collection and Pathogens Implicated in VAP

Regarding the methodology for obtaining lung samples, once again the neonatal patient represents a significant challenge. In this group of patients, less invasive techniques are usually used, such as direct tracheal aspiration (TA) in intubated patients, which does not allow differentiation between colonization and infection, with almost universal bacterial growth being found when the endotracheal tube has been in place for more than 10 days [71]. In adults, bronchoscopic bronchoalveolar lavage (BAL) and protected brush are very reliable techniques as they avoid contamination of the samples; however, they are not applicable to newborns due to the small diameter of the endotracheal tube. On the contrary, the use of blind-protected invasive sampling techniques is feasible and seems to minimize the sample contamination [61]. In this study, carried out with blind-protected BAL, the most frequently isolated pathogens were *Pseudomonas aeruginosa* and *Staphylococcus aureus*, with the isolate being polymicrobial in 16.7% of the cases [61]. Other studies have shown predominance of Gram-negative rods, especially *Klebsiella pneumoniae*, *Escherichia coli*, *Enterobacter* spp., *Acinetobacter* spp., *Citrobacter* spp., *Acinetobacter baumanii*, and *Stenotrophomonas maltophilia* [72,73,74,75,76], as well as Gram-positive cocci, such as *Coagulase negative Staphilococcus*, *Staphilococcus aureus*, *Enterococcus*, and *Group B Streptococcus* [74,77]. It is important to highlight that, when TA are direct through an endotracheal tube, polymicrobial isolation can reach up to 58% [72].

Given the difficulty of differentiating pneumonia from bacterial colonization, specific biomarkers of VAP have been sought in the adult population, such as the presence of intracellular microorganisms, the detection of elastin fibers, the test for antibody-coated bacteria, the level of endotoxins in the BAL fluid, the local production of interleukin-8, the levels of lactate dehydrogenase and the decrease in surfactant protein A, leading to poor results. Perhaps the most validated technique featuring greater specificity, as long as the patient does not receive prior antibiotic therapy, is the search for intracellular bacteria in polymorphonuclear leukocytes or macrophages. However, this technique requires a considerable time effort on the part of the microbiologist, and also requires the performance of BAL, which is not always available or free of risks for the patient [78].

As mentioned before, in the neonatal patient, Cernada et al. [60] proposed the use of BAL with catheters with blind protection, although it is still necessary to carry out comparative trials versus traditional TA for culture, as well as to identify reliable biomarkers of lung infection to diagnose VAP. In their excellent review, they discuss the potential usefulness of various markers still under investigation, such as Procalcitonin, Cytokines such as IL-1, IL-6, IL-8, IL-10, and TNF-α, the Soluble Form of the Triggering Receptor Expressed on Myeloid Cells (s-TREM), and plasminogen activation inhibitor-1 (PLA-1). Of them, in a study from Srinivasan et al. [79], elevated levels of PLA-1 had the strongest association with a clinical diagnosis of VAP and were the best biomarker to differentiate VAP from colonization in a pediatric population. More recently, Pinilla-González et al. concluded that TNF-α in BAL fluid and glutathione sulfonamide in both BAL fluid and TA were associated with VAP in preterm newborns, making them useful as early biomarkers of VAP, although further studies with more patients are needed to confirm these results [80]. Finally, as in adults, it seems Gram stain studies in TA samples are the most promising. In the study by Katayama et al. [81] in VLBW infants, a sensitivity of 82 and 100%, and a specificity of 100 and 82%, was found for Gram-positive and Gram-negative VAP, respectively. Furthermore, initial antibiotic therapy based on TA with Gram staining was effective in 96% of cases. More recently, Ergenekon et al. observed in a TA fluid cytospin sample (a monolayer of cells by rotation and centrifugal acceleration) from a patient with severe BPD and VAP, abundant intraepithelial bacteria, which were no longer detectable after a course of intravenous antibiotics, whereas TA fluid cultures grew the same microorganisms before and after treatment [82].

### 3.3. Management

The proper management of VAP is based on two fundamental issues: prevention and careful treatment, avoiding overtreatment as well as not treating established cases. Most clinicians choose to treat most suspected cases because of the potentially high risk of undertreatment. A basic aspect of the prevention of healthcare-associated infections is the protection and promotion of the establishment of an appropriate microbiota that competes with pathogens for the colonization of the corresponding ecological niches. In this regard, it is known that early, inappropriate, or excessive use of broad-spectrum antibiotics is significantly associated with the emergence of multidrug-resistant pathogens [83]. In addition to this, different sets of measures have been developed that, despite the limitation of not having clearly established and universal diagnostic criteria, have shown the effectiveness in reducing VAP in local settings. A summary of the potential measures suggested by different authors and adapted from bundles developed for the prevention of VAP in adults is shown in Table 4 [84].

Otherwise, specific treatment is aimed at broad-spectrum antibiotic coverage based on the predominant pathogens in the units, with subsequent de-escalation based on bacterial isolation and the antibiogram. However, it is necessary to take into account that neonatal VAP does not usually progress to systemic bacteremia or rapid clinical deterioration, and it is accompanied by a lower mortality rate compared to neonatal bacteremia or VAP in adults [85]. Therefore, it is worth reconsidering the need to use broad-spectrum antibiotics to treat neonates with VAP without concurrent bacteremia.

Another important aspect of the treatment has to do with the replacement of the theoretically most favorable microbiota that must occupy a certain niche to compete with pathogens. The intestinal microflora has anti-inflammatory, antioxidant and analgesic properties, and produces vitamins which protect the intestine against the actions of pathogenic bacteria that can cause chronic diseases [86]. Given the increasing antibiotic resistance in recent times, the investigation of the therapeutic potential of the intestinal microbiota is of paramount importance. At present there are no conclusive studies that allow us to establish a specific guideline with a sufficient level of evidence, but the use of probiotics and prebiotics has been proposed for years in an attempt to restore the intestinal microbiome. Initial studies administering *Lactobacillus* and/or *Bifidobacterium* in premature neonates seemed promising for preventing severe necrotizing enterocolitis and mortality [87], although, as discussed above, these results were not subsequently confirmed, especially in extremely premature infants [33]. In a model of newborn rhesus macaques, Stevens et al. [23] observed that exposure to antibiotics early in life promoted inflammatory changes and made them more susceptible to bacterial pneumonia. The transfer of fecal microbiota allowed partial correction of general immune maladaptation and protection against severe pneumonia. The authors conclude that their findings show the importance of the gut microbiota in programming lung immunity and support the idea that the gut microbiota promotes balance between pathways that drive tissue repair and inflammatory responses associated with clinical recovery of infection in babies. The possibility of using immunomodulatory drugs or oral or nebulized probiotics has also been suggested [21,88,89]. Some experimental studies on mice have already shown that nasal administration of probiotic bacteria can protect against respiratory viral infections [90,91]. In humans, a study carried out almost a decade ago in adult volunteers showed that nasal inoculation of *Neisseria lactamica* displaced *Neisseria meningitidis*, also indicating that this inhibition of meningococcal carriage could be even more potent than after glycoconjugate meningococcal vaccination. The authors conclude that *Neisseria lactamica* or its components could be a novel bacterial medicine to suppress meningococcal outbreaks [92]. Similarly, a study that evaluated the prophylactic use of probiotics for the prevention of VAP in children admitted to a high-risk pediatric ICU (where baseline VAP rates were high) showed a 77% decrease in the incidence of VAP. The intervention was found to be safe and the results seemed promising in this setting [93]. However, the recent PROSPECT trial on the prevention of severe pneumonia and endotracheal colonization by intratracheal administration of the probiotic *L rhamnosus GG* compared with a placebo in adults resulted in no significant difference in the development of ventilator-associated pneumonia [94]. Therefore, more studies are still necessary in this field.

## 4. Conclusions

Gaining knowledge of the microbiome of the respiratory system, especially at the distal or pulmonary level, in newborns has only just begun. The importance of the maternal microbiota, and its influence even before birth, is now well recognized. The type of birth and feeding of the baby, as well as the environment in which they occur, are essential for establishing the individual’s microbiota pattern. The use of antibiotics administered to both the mother and newborn are among the factors that most affect the establishment and development of a healthy and protective microbiota. The dysbiosis that follows its administration has been associated with several health problems in the short and long term. In the present review, we focused on the development of VAP, especially in intubated preterm newborns. Although precise diagnostic criteria are far from being achieved, the appropriate use of antibiotics in the perinatal period, as well as avoiding endotracheal intubations or shortening invasive mechanical ventilation times, are among the most relevant preventive measures. The potential use of oral or aerosol probiotics to prevent or treat complications derived from dysbiosis constitutes a field of great interest in modern perinatology.

## Figures and Tables

**Table 2:** Diagnostic criteria for ventilator-associated pneumonia (VAP) in infants aged 1 year or younger (Adapted from CDC, 2024 [3]).

**Imaging**	Patient without underlying diseases 1 or more (or with underlying diseases 2 or more) imaging test results with one of the following: New and persistent or progressive and persistent − Infiltrate − Consolidation − Cavitation − Pneumatoceles
**Signs and Symptoms**	Worsening gas exchange (for example, O_2_ desaturations [for example, pulse oximetry < 94%], increased oxygen requirements, or increased ventilator demand) AND at least THREE of the following: Temperature instability Leukopenia (≤4000 WBC/mm^3^) or leukocytosis (≥15,000 WBC/mm^3^) and left shift (≥10% band forms) New onset of purulent sputum, or change in character of sputum, or increased respiratory secretions, or increased suctioning requirements Apnea, tachypnea, nasal flaring with retraction of chest wall, or nasal flaring with grunting Wheezing, rales, or rhonchi Cough Bradycardia (<100 beats/min) or tachycardia (>170 beats/min)

**Table 3:** Microbiological criteria for ventilator-associated pneumonia (Adapted from CDC, 2024 [3]).

**Laboratory**	At least one of the following: Organism identified from blood, ruled out other sources of infection Organism identified from pleural fluid Positive quantitative culture or corresponding semi-quantitative result from minimally contaminated LRT specimen (specifically, BAL, protected specimen brushing, or endotracheal aspirate) ≥5% BAL-obtained cells contain intracellular bacteria on direct microscopic exam Positive quantitative culture or corresponding semi-quantitative result of lung tissue Histopathologic exam shows at least one of the following: - Abscess formation or foci of consolidation with intense PMN accumulation in bronchioles and alveoli - Evidence of lung parenchyma invasion by fungal hyphae or pseudo-hyphae
**Threshold values for cultured specimens according to the collection technique**	
**Specimen collection/technique**	**Values**
Lung tissue	≥10^4^ CFU/g tissue
Bronchoscopically (B) obtained specimens	
Bronchoalveolar lavage (B-BAL)	≥10^4^ CFU/ml
Protected BAL (B-PBAL)	≥10^4^ CFU/ml
Protected specimen brushing (B-PSB)	≥10^3^ CFU/ml
Nonbronchoscopically (NB) obtained (blind) specimens	
NB-BAL	≥10^4^ CFU/ml
NB-PSB	≥10^3^ CFU/ml
Endotracheal aspirate (TA)	≥10^5^ CFU/ml

CFU = colony forming units, g = gram, ml = milliliter.

**Table 4:** Interventions to prevent neonatal VAP, adapted from adults bundles [84].

Hand hygiene: o Meticulous hand hygiene before and after patient contact for oral care and handling respiratory equipment and supplies. o Wear gloves when handling ventilator condensate and other respiratory/oral secretions. Endotracheal Intubation: o Use a new, sterile ET for each intubation attempt. o Ensure the tube does not contact environmental surfaces before insertion. o Use a sterilized laryngoscope. o At least two NICU staff members for ET tape or repositioning. Suctioning practices: o Clear secretions from the posterior oropharynx prior to: - ET manipulation; - Patient repositioning; - Extubation; - Reintubation. o Use closed ET suction system. o Suction ET tube “as-needed” and avoid using normal saline. Feeding: o Prevent gastric distention: - Avoid bolus feedings. - Monitor gastric residuals every 3–4 h. - Monitor abdominal girth. Positioning: o Use side-lying position as tolerated. o Keep the head of bed elevated 15°–30° as tolerated. o Use left lateral positioning after feedings, as tolerated. Oral care: o Provide oral care within 24 h after intubation and then: - Every 3–4 h; - Prior to reintubation as time allows; - Prior to orogastric tube insertion. o Use sterile water, mother’s milk, or approved pharmaceutical oral care solution. o Avoid petroleum-based moisturizer if open wounds are present, as well as moisturizers containing alcohol and lemon–glycerine compounds. Respiratory equipment o Use separate suction catheters, tubing, and canisters for oral and tracheal suction. o Change in-line suction catheter system only when soiled or otherwise indicated. o Store reusable resuscitation bag and oral suction catheters outside the bed in a clean, non-sealed plastic bag between use. o Drain ventilator condensate from the patient every 2–4 h and before repositioning. o Avoid unnecessary disconnection of the ventilator circuit. o Change ventilator equipment when visibly soiled or mechanically malfunctioning. o Use heated ventilator circuits.

ET, endotracheal tube; NICU, neonatal intensive care unit.

## Data Availability

No new data have been generated in this review.
